# Rhinovirus-induced IFNβ expression is NFκB-dependent and regulated by the macrophage microenvironment

**DOI:** 10.1038/s41598-019-50034-1

**Published:** 2019-09-16

**Authors:** Mandy Menzel, Joakim Kosinski, Lena Uller, Hamid Akbarshahi

**Affiliations:** 1 Respiratory Immunopharmacology, Department of Experimental Medical Science, Lund University, Lund, Sweden; 2 Respiratory Medicine and Allergology, Department of Clinical Sciences, Lund, Lund University, Lund, Sweden

**Keywords:** Viral infection, RIG-I-like receptors

## Abstract

Macrophages play an important role in asthma pathogenesis both in the inflammatory and resolution phase of the disease. Macrophages can acquire different polarisation states dependent on their microenvironment. It is yet unclear through which mechanism the microenvironment affects the anti-viral response in macrophages. We hypothesized that the macrophage microenvironment regulates rhinovirus-induced IFNβ expression. Murine bone marrow-derived monocytes and human differentiated THP-1 cells were stimulated with M-CSF or GM-CSF and IFNγ or IL-4/IL-13, respectively, to mimic a Th1 or Th2 environment. Macrophages were infected with rhinovirus and gene and protein levels of IFNβ and pattern recognition receptor expression were measured. In subsequent experiments an IκB kinase inhibitor was used to study the involvement of NFκB. Both murine and human M1-like macrophages exhibited higher levels of IFNβ and pattern recognition receptors after rhinovirus infection than M2-like macrophages. Blockage of NFκB resulted in a lower expression of rhinovirus-induced IFNβ in human M1-like macrophages while inducing a higher expression in M2-like macrophages, suggesting that the interferon response towards viral infection was mediated by NFκB. These findings could contribute to a better understanding of mechanisms causing reduced anti-viral responses at viral-induced exacerbations in asthma.

## Introduction

Asthma is a chronic disease of the airways that presents as wheezing, shortness of breath and chest tightness. Characteristic of asthma is a reversible airway obstruction caused by bronchial smooth muscle constriction and inflammation in the lungs^1,2^. Environmental factors, such as allergens, play a fundamental role in the development of allergic asthma. Allergic asthma is typically associated with a Th2 type immune response, characterised by the production of IL-4, IL-13 and IL-5, and elevated levels of eosinophils^3^.

Macrophages are one of the most abundant leukocytes found in the respiratory tract and are key modulators and effector cells of the immune response. Two distinct macrophage populations exist in the lung, alveolar macrophages, and interstitial macrophages. Alveolar macrophages are involved in inflammatory responses, while interstitial macrophages are important for lung homeostasis^4^. Macrophages are recruited to the site of injury, aiding in the clearance of pathogens and, by cooperation with immune cells, constrain and repair tissue damage. The function and phenotype of macrophages can vary due to their versatile and plastic nature^5^. Plasticity is controlled by changes in the environment and alveolar macrophages can be broadly divided into two different phenotypes: M1 and M2^6^. M1 phenotype or the classical activation pathway is initiated upon Toll-like receptor (TLR) stimulation by microbial products and/or in presence of IFNγ, activating a Th1 type immune response. In addition, other pro-inflammatory cytokines, such as TNFα and IL-1β, can act as M1 stimuli. M2 phenotype or the alternative activation pathway plays a role in allergy and anti-parasitic defences and can be stimulated by the Th2 cytokines IL-4 and IL-13. M2 macrophages are efficient producers of anti-inflammatory cytokines^7^. Dependent on the microenvironment macrophages can switch between these polarization phenotypes.

Besides these cytokine regulators, transcription factors can also control macrophage polarisation. The nuclear factor kappa-light-chain-enhancer of activated B cells (NFκB/Rel) transcription factor family initiates inflammatory cytokine responses. Alveolar macrophages have been shown to activate the NFκB pathway and promote the release of TNFα upon rhinovirus infection^8^.

Transcription factor activation is regulated by IκB kinase β (IKKβ) through phosphorylation of IκBα. IKKβ deficient macrophages produce lower levels of IFNβ upon group B streptococcus infection^9^.

Numbers of M2 macrophages are higher in asthmatic patients^10^ and in mice with allergic airway inflammation^11,12^ compared with control subjects. Further, asthma severity has been shown to be associated with increased M2 macrophage count^10^ and Ford, *et al*.^13^ demonstrated in a mouse model of allergic lung inflammation that M2 macrophages are drivers of inflammation by recruiting eosinophils.

Asthma exacerbation is an episodic worsening of the disease that is mediated by increased expression of inflammatory cytokines and chemokines. Asthma exacerbations are often triggered by infections with respiratory viruses, particularly rhinovirus^14^. Rhinoviruses can be largely divided into clades A, B, and the newly discovered clade C. Viruses of clade C utilize the cadherin-related family member 3 (CDHR3) for cell entry. Viruses of clade A and B are divided into two groups: major group rhinoviruses bind to intercellular adhesion molecule 1 (ICAM-1) and minor group rhinoviruses utilize low density lipoprotein (LDL) receptor for cell entry. Cell entry is followed by viral replication. Upon replication, dsRNA is formed which is recognized by pattern recognition receptors such as the endosomal toll-like receptor 3 (TLR3) and the cytoplasmic retinoic acid-inducible gene I (RIG-I) like helicases RIG-I and melanoma differentiation-associated protein 5 (MDA5)^15,16^. This interaction initiates downstream signalling to activate the production of interferons that are central mediators of anti-viral defences. It has been demonstrated that asthmatic patients respond towards viral infection with a deficient production of interferon β (IFNβ), resulting in greater viral burden in these individuals^17^. In cultured macrophages, induction of interferons was found to be stimulated by rhinovirus infection^12,18^. Constitutive interferon expression is crucial for the phagocytotic potential of macrophages^19^. A recent study found that bronchoalveolar lavage cells of asthmatic subjects, constituting of nearly 95% macrophages, show a reduced interferon response towards rhinovirus infection compared to healthy individuals^20^.

While the involvement of macrophages in allergic inflammation is well known not many studies have investigated a possible role of macrophages on outcomes of viral-induced asthma exacerbations. Here we demonstrate that the polarization phenotype affects the production of viral-induced IFNβ and pattern recognition receptor expression both in human and murine derived macrophages. We further demonstrate that NFκB is an important regulator of IFNβ production.

## Results

### GM-CSF differentiated murine bone marrow-derived macrophages have reduced expression of IFNβ upon RV1B infection

**Figure 1:**
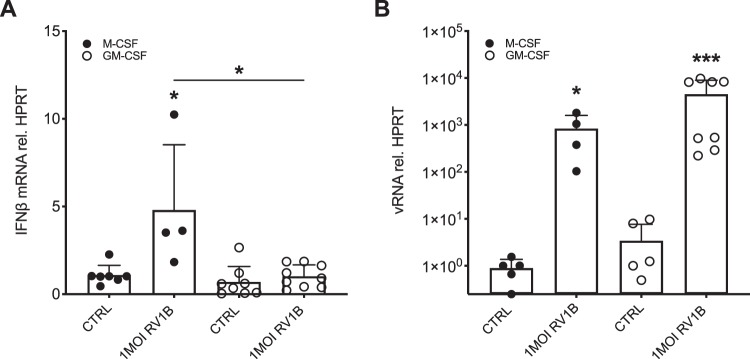
Bone marrow-derived murine macrophages differentiated with GM-CSF have reduced expression of IFNβ upon RV infection and a trend towards a higher expression of viral load. Bone marrow-derived murine monocytes were differentiated to macrophages by stimulation with either GM-CSF or M-CSF followed by infection with RV1B for 24 h. Gene expression of IFNβ (**A**) and vRNA (**B**) was measured by real-time PCR and data is presented as mean ± standard deviation fold change of untreated M-CSF stimulated macrophages relative to HPRT expression. Comparison of different groups was performed by Kruskal-Wallis with Wilcoxon post-testing. *p < 0.05, **p < 0.01. ***p < 0.001 vs. own CTRL if not stated otherwise. Data from 6 independent experiments.

Murine bone marrow-derived macrophages were differentiated by stimulation with either GM-CSF or M-CSF. Infection with RV1B induced IFNβ gene expression in differentiated murine macrophages. Induction of IFNβ was lower in macrophages stimulated with GM-CSF than in M-CSF stimulated macrophages (p < 0.05; Fig. 1A). Associated with this was a trend towards higher viral load in GM-CSF stimulated macrophages compared to macrophages stimulated with M-CSF after rhinovirus infection (Fig. 1B).

### Expression of RIG-I like helicases is reduced upon RV infection in GM-CSF stimulated murine bone marrow-derived macrophages

**Figure 2:**
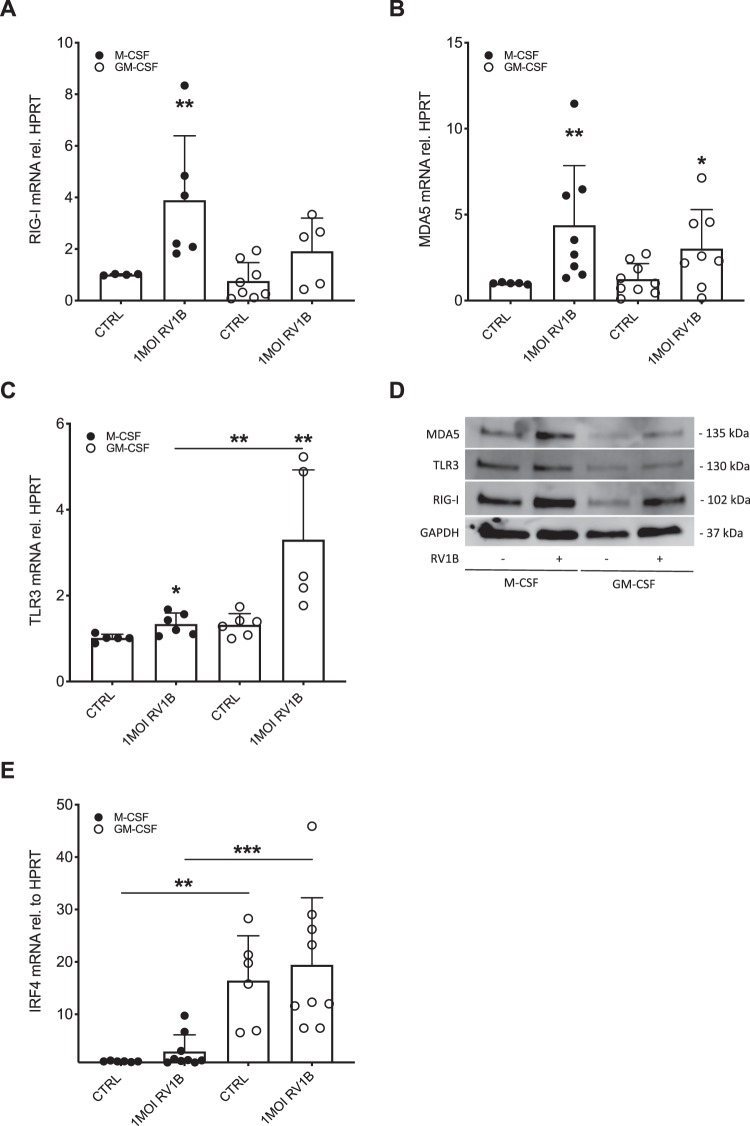
Bone marrow-derived murine macrophages differentiated with GM-CSF show a trend towards a reduced expression of pattern recognition receptors upon RV infection while exhibiting increased expression of IRF4. Bone marrow derived monocytes were differentiated to macrophages by stimulation with either GM-CSF or M-CSF followed by infection with RV1B for 24 h. Gene expression of RIG-I (**A**), MDA5 (**B**), TLR3 (**C**) and IRF4 (**E**) was measured by real-time PCR and data is presented as mean ± standard deviation fold change of untreated M-CSF stimulated macrophages relative to HPRT expression. Comparison of different groups was performed by Kruskal-Wallis with Wilcoxon post testing. *p < 0.05, **p < 0.01, ***p < 0.001 vs. own CTRL if not stated otherwise. Data from 6 independent experiments. A representative Western Blot image of TLR3, RIG-I and MDA5 protein is shown (**D**). Blots were stripped after incubation with anti-TLR3 mAb and successively reprobed with anti-RIG-I mAb and anti-MDA5 mAb. Blots were run on the same gel and were not cropped.

Rhinovirus-induced IFNβ expression is mediated by pattern recognition receptors, most prominently TLR3 and the RIG-I like helicases RIG-I and MDA5. Expression of the RIG-I like helicases RIG-I and MDA5 showed a trend towards lower expression after rhinovirus infection in murine macrophages stimulated with GM-CSF compared to those stimulated with M-CSF (Fig. 2A, B) as confirmed on protein level (Fig. 2D). In contrast, gene expression of TLR3 was significantly higher in GM-CSF stimulated murine macrophages (p < 0.01; Fig. 2C), while protein levels of TLR3 were reduced in GM-CSF stimulated murine macrophages (Fig. 2D).

### GM-CSF differentiated murine bone marrow-derived macrophages display a higher baseline expression of IRF4

It has long been thought that GM-CSF primes monocytes towards an M1 phenotype, while M-CSF primes them to a M2 phenotype^21^. However, newer research shows that stimulation with GM-CSF can lead to a more “M2-like” phenotype showing high expression of IRF4^22^. To establish whether in our setting GM-CSF primes macrophages towards a “M1-like” or a “M2-like” phenotype we investigated IRF4 expression. The expression of IRF4 was significantly higher at baseline (p < 0.01) in GM-CSF primed murine macrophages and was not further elevated by RV infection (Fig. 2E).

### M2-primed macrophages show reduced expression of IFNβ but no change in viral load after infection with RV16

**Figure 3:**
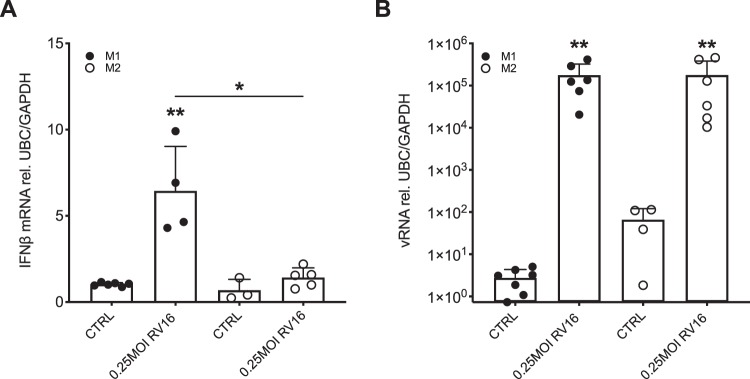
M2 differentiated THP-1 cells display reduced expression of IFNβ upon RV infection while the viral load is not affected. THP-1 cells were differentiated towards an M1 and M2 phenotype by stimulation with IFNγ and IL-4/IL-13, respectively. Macrophages were infected with RV16 for 24 h. Gene expression of IFNβ (**A**) and vRNA (**B**) was measured by real-time PCR and data is presented as mean ± standard deviation fold change of untreated M1 differentiated macrophages relative to UBC/GAPDH expression. Comparison of different groups was performed by Kruskal-Wallis with Wilcoxon post-testing. *p < 0.05, **p < 0.01 vs. own CTRL if not stated otherwise. Data from 6 independent experiments.

Human THP-1 cells were differentiated by treatment with PMA into a resting macrophage state (M0). M0 cells were further differentiated by treatment with either IFNγ into M1 phenotype or with a combination of IL-4 and IL-13 into M2 phenotype^23^. This was followed by infection with 0.25 MOI RV16. Infection with RV16 resulted in a significant increase of IFNβ gene expression in M1-primed macrophages (p < 0.01), while there was no induction of IFNβ gene expression after RV16 infection in M2-primed macrophages (Fig. 3A). Interestingly, there was no difference in viral load between M1- and M2-primed macrophages (Fig. 3B).

### Pattern recognition receptor expression is reduced in M2-primed macrophages

**Figure 4:**
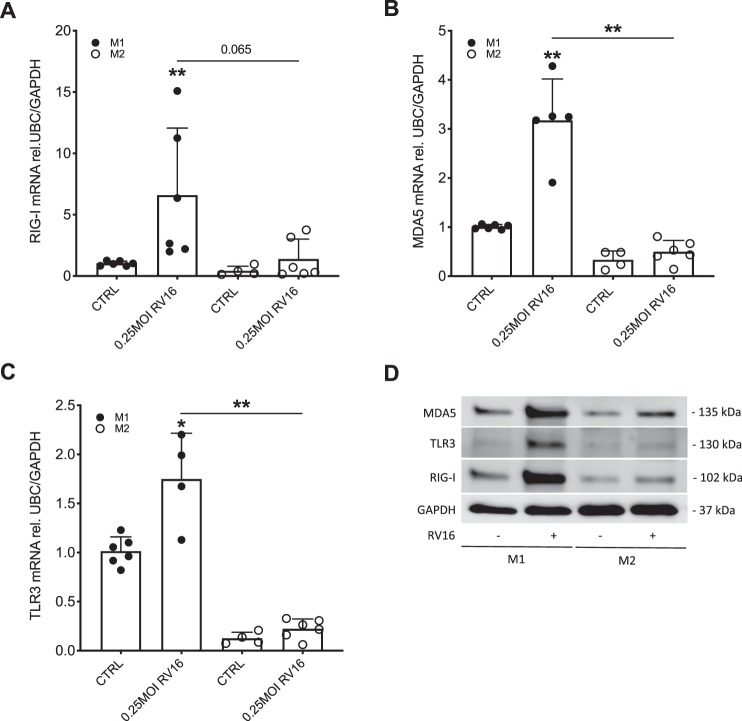
In M2 differentiated THP-1 cells expression of pattern recognition receptors is decreased upon RV infection. THP-1 cells were differentiated towards an M1 and M2 phenotype by stimulation with IFNγ and IL-4/IL-13, respectively. Macrophages were infected with RV16 for 24 h. Gene expression of RIG-I (**A**), MDA5 (**B**) and TLR3 (**C**) was measured by real-time PCR and data is presented as mean ± standard deviation fold change of untreated M1 differentiated macrophages relative to UBC/GAPDH expression. Comparison of different groups was performed by Kruskal Wallis with Wilcoxon post-testing. *p < 0.05, **p < 0.01 vs. own CTRL if not stated otherwise. Data from 6 independent experiments. A representative Western Blot image of TLR3, RIG-I and MDA5 protein is shown **(D)**. Blots were stripped after incubation with anti-TLR3 mAb and successively reprobed with anti-RIG-I mAb and anti-MDA5 mAb. Blots were run on the same gel and were not cropped.

As differentiation of macrophages affected their interferon response towards rhinovirus infection, we intended to investigate if the expression of pattern recognition receptors was similarly affected. Comparably to IFNβ expression, infection with RV16 induced gene expression of TLR3, RIG-I, and MDA5 in M1-primed macrophages but there was no induction of these pattern recognition receptors in M2-primed macrophages (Fig. 4A–C). This could also be confirmed on the protein level (Fig. 4D).

### Expression of RV-induced IFNβ and PRRs is reduced by inhibition of NFκB signalling in M1-primed macrophages while IFNβ expression is increased in M2-primed macrophages

**Figure 5:**
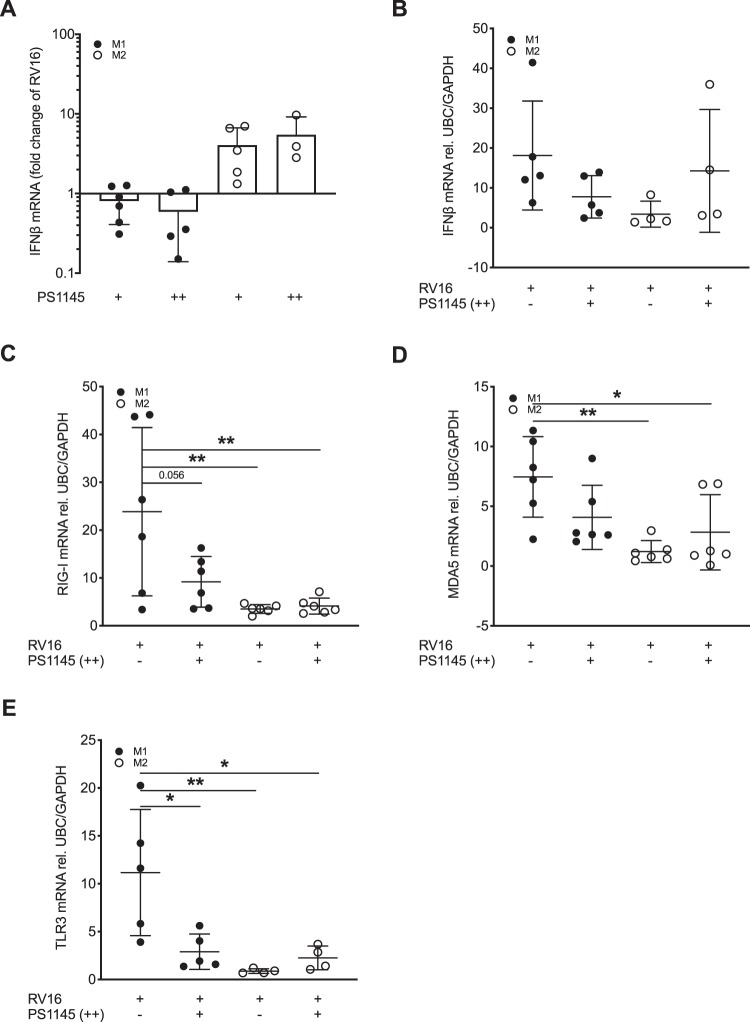
Inhibition of NFκB signalling reduces RV-induced expression of IFNβ and pattern recognition receptors in M1 differentiated THP-1 cells while increasing expression of IFNβ in M2 differentiated THP-1 cells. THP-1 cells were differentiated towards an M1 and M2 phenotype by stimulation with IFNγ and IL-4/IL-13, respectively. Macrophages were infected with RV16 for 24 h. At RV infection (+) or at the time of cytokine stimulation and after RV infection (++) the IκB kinase inhibitor PS1145 was added. Gene expression of IFNβ (**A, B**), RIG-I (**C**), MDA5 (**D**) and TLR3 (**E**) was measured by real-time PCR and data is presented as mean ± standard deviation fold change of untreated M1 differentiated macrophages relative to UBC/GAPDH expression (**B**–**E**) or expressed as fold change of RV infected macrophages relative to UBC/GAPDH expression **(A)**. Comparison of different groups was performed by one-way ANOVA with Tukey post- testing. *p < 0.05, **p < 0.01. Data from 4 independent experiments.

NFκB is a key transcription factor of M1 macrophage activation^24^ and crucial for early induction of anti-viral responses^25^. In order to investigate how a lack of NFκB affects anti-viral responses in polarised macrophages, cells were incubated with the IκB kinase inhibitor PS1145 either after RV infection or at the time of macrophage differentiation as well as after RV infection. In M1-primed macrophages inhibition of NFκB signalling decreased RV-induced IFNβ expression, while enhancing RV-induced IFNβ expression in M2-primed macrophages (Fig. 5A). The timing of exposure to PS1145 (before or after macrophage polarisation) did not affect the observed effects on IFNβ expression. The blockage of NFκB signalling partially restored the reduced IFNβ response in M2-primed macrophages (Fig. 5B). While exposure to PS1145 reduced RV-induced expression of RIG-I, MDA5, and TLR3 in M1-primed macrophages, it did not alter their expression in M2-primed macrophages (Fig. 5C–E), suggesting that lack of NFκB alters IFNβ expression independent of pattern recognition receptors in M2-primed macrophages.

## Discussion

Asthma exacerbations are often caused by infection with respiratory viruses, especially rhinoviruses, and result in worsening of asthma symptoms. It is not fully understood why infection with rhinovirus, the common cold virus, causes severe illness in asthmatics and even less is known how the macrophage activation state affects anti-viral responses. There has been a debate about whether or not rhinovirus infects and replicates in macrophages. Laza-Stanca, *et al*.^8^ have previously shown that rhinovirus can replicate in THP-1 derived macrophages and mount robust activation of NFκB. Here we demonstrate that M2-primed macrophages, as prominent during allergic inflammation, show no production of RV-induced IFNβ compared to M1-primed ones. This was associated with a lower expression of pattern recognition receptors in M2 macrophages. In addition, blocking of the NFκB signalling pathway reduced RV-induced IFNβ expression in M1-primed macrophages, while increasing IFNβ in M2-primed macrophages.

It is well established that Th1 and Th2 cytokines polarize macrophages broadly into M1 and M2. It has been long thought that GM-CSF polarizes macrophages into an M1-like phenotype while M-CSF facilitates polarization towards an M2-like phenotype. This concept is now under debate: IRF4 is a well-known inducer of M2 associated genes and M2 phenotype polarization^26^. However, GM-CSF has been shown to induce IRF4 expression in human monocytes^22,27^ and in murine bone marrow-derived macrophages^22,28^. This is in accordance with our findings of a higher baseline expression of IRF4 in GM-CSF stimulated murine bone marrow-derived macrophages compared to M-CSF stimulated ones. Infection of GM-CSF and M-CSF primed murine macrophages with RV1B only induced IFNβ expression in M-CSF primed macrophages, suggesting that viral-induced expression of IFNβ is deficient in GM-CSF primed murine macrophages. This was associated with a trend towards higher viral load in GM-CSF primed murine macrophages. This is of interest, as the expression of GM-CSF is enhanced in bronchoalveolar lavage fluid, sputum and bronchial biopsies of asthmatics^29,30^.

In human macrophages priming with IFNγ towards an M1 phenotype resulted in RV infection induced IFNβ expression, while there was no induction of IFNβ expression in human macrophages primed with IL-4 and IL-13 towards an M2 phenotype. Our findings are in agreement with previous reports of interferon deficiency in asthmatics^17,20^, for which higher numbers of M2 primed macrophages in the lungs are recorded^10^. Despite differences in IFNβ expression, we did not observe alterations in viral load between the different polarisation phenotypes. However, it cannot be excluded that these might come apparent at another time point.

The transcription factor NFκB is known to be involved both in macrophage polarisation^24^ and induction of an early anti-viral interferon response^25^. As we observed clear differences in rhinovirus-induced levels of IFNβ expression between M1-like and M2-like macrophages, we hypothesised that these differences could be due to differential NFκB activation. Human macrophages were incubated with an IκB kinase inhibitor at the time of induction of polarisation and/or after RV infection. In M1-like macrophages inhibition of NFκB signalling reduced RV-induced IFNβ expression, while in M2 macrophages RV-induced IFNβ expression was enhanced. It has been previously shown that blockage of NFκB impairs RV-induced IFNβ expression in monocyte-derived macrophage^31^, and murine macrophages deficient in IKKβ produce lower levels of IFNβ when exposed to a Th1 stimulus such as group B streptococcus infection^9^. These findings suggest that in macrophages induction of IFNβ is predominantly mediated by NFκB. This could also explain the minimal induction of IFNβ expression by rhinovirus in M2-primed macrophages, as here NFκB signalling is repressed by enhanced expression of the NFκB subunit p50^32^.

In many cell types the transcription factor IRF3, which is constitutively expressed, is, once phosphorylated, involved in inducing IFNβ expression^33^. IRF3 signalling has been demonstrated to be up-regulated in M2 macrophages^34,35^. As we found RV-induced IFNβ expression only partially reduced by inhibition of NFκB in M1-primed macrophages, it could be speculated that IRF3 contributes at least in part to the IFNβ response in these cells. In contrast, in M2-primed macrophages, where IRF3 levels are enhanced, lack of NFκB could result in a solely IRF3-driven IFNβ expression. However, further investigation of a possible NFκB-IRF3-crosstalk is warranted.

Pattern recognition receptors such as TLR3 and the RIG-I like helicases MDA5 and RIG-I recognize viral dsRNA and facilitate downstream signalling to mount an inflammatory response and production of interferons, leading to an anti-viral state with the induction of interferon-stimulated genes^36^. Here, both murine M-CSF stimulated and human M1-like macrophages had a higher RV-induced expression of RIG-I like helicases than murine M-CSF stimulated and human M2-like macrophages both on gene and protein level, tightly following the expression pattern of IFNβ. These findings are in line with a recent study by Rajput, *et al*.^37^ who observed higher responsiveness to rhinovirus infection in M1-primed macrophages compared to M2-primed macrophages, which was associated with higher expression of MDA5. In our study, RV-induced expression of TLR3 was reduced in human M2-like macrophages compared to M1-like ones. In murine-derived macrophages, RV-induced TLR3 gene expression was higher in GM-CSF stimulated macrophages, while protein expression was lower in GM-CSF stimulated macrophages than in M-CSF stimulated ones. This discrepancy could be due to time differences in gene and protein expression.

When NFκB signalling was blocked in M1-like macrophages RV-induced expression of pattern recognition receptors was diminished, following the expression pattern of IFNβ. However, in M2-like macrophages, where lack of NFκB increased RV-induced IFNβ expression, pattern recognition receptor expression was not altered, suggesting that the effects of NFκB signalling blockage were not mediated by pattern recognition receptors and were likely downstream of these.

In conclusion, we demonstrate that polarisation of macrophages by a Th1 respective Th2 cytokine environment affects their ability to mount an anti-viral response towards rhinovirus infection. The majority of asthma is associated with a Th2 response and deficiency in interferon-producing monocytes/macrophages during rhinovirus infection has been observed in asthmatics^38^. Here, we show that reduced levels of IFNβ produced by M2-like macrophages upon viral infection could contribute to this deficient interferon response observed in asthmatics. Our observation of NFκB differentially regulating anti-viral responses in polarised macrophages needs further investigation.

## Methods

### Isolation of bone marrow derived monocytes (BMDM)

C57BL/6 mice of 6–8 weeks of age were euthanized by cervical dislocation. Animal experiments were performed in accordance with standard ethical guidelines and approved by the regional laboratory animal ethics committee in Malmö/Lund (Permit No. M36-13). Femoral and tibial bones were dissected. Skin and excess muscle tissue were removed. Femur and tibia were separated by a cut at the knee joint and bone marrow was flushed with 10 ml of phosphate-buffered saline (PBS; Life Technologies, Carlsbad, CA, USA) supplemented with 2% heat-inactivated fetal bovine serum (HI-FBS; Life Technologies, Carlsbad, CA, USA). Bone marrow was passed through 70 µm nylon cell strainer (Fisher Scientific, Lund, Sweden), centrifuged and resuspended and incubated in ammonium-sodium-chloride lysis buffer (ACK; Life Technologies, Carlsbad, CA, USA) for 5 min to lyse red blood cells. Cells were then washed with PBS with 2% HI-FBS, centrifuged and resuspended in RPMI 1640 culture medium supplemented with 10% HI-FBS and 1% penicillin-streptomycin.

For additional experiments, the human monocytic leukemia cell line THP-1 (InvivoGen, San Diego, CA, USA) was cultured in RPMI 1640 culture medium supplemented with 10% HI-FBS and 1% penicillin-streptomycin.

### Differentiation of monocytes to macrophages

For differentiation, BMDM were treated with either 50 ng/ml macrophage-colony stimulatory factor (M-CSF; PeproTech, Stockholm, Sweden) or 50 ng/ml granulocyte macrophage-colony stimulatory factor (GM-CSF; PeproTech, Stockholm, Sweden) for 7 days and non-adherent cells were washed away with PBS. Adherent cells were considered macrophages.

For differentiation THP-1 cells were treated with 50 ng/ml phorbol-12-myristate-13-acetate (PMA; Sigma Aldrich, Stockholm, Sweden) for 48 h to obtain a macrophage-like state and then rested for an additional 48 h in culture medium to obtain the resting state of macrophages (M0). Resting-state THP-1 macrophages were further differentiated towards either a M1 phenotype (20 ng/ml IFNγ; R&D Systems, Abingdon, UK) or a M2 phenotype (20 ng/ml IL-4 and 20 ng/ml IL-13; R&D Systems, Abingdon, UK) as previously described^23^.

### Stimulation of macrophages

Primed human macrophages were infected with rhinovirus RV16, a major group rhinovirus that utilizes ICAM-1 for cell entry, for 24 h. Primed mouse macrophages were infected with RV1B, a minor group rhinovirus that utilizes LDL receptor for cell entry, as mice do not express ICAM-1 receptor^39^. Cells were infected with rhinovirus for 1 h at room temperature while shaking. Then the virus was removed and fresh medium was added to the cells. Cell lysates were collected 24 h post rhinovirus infection for subsequent gene expression analysis by quantitative real-time PCR and protein expression analysis by western blot.

In subsequent experiments, human macrophages were treated with the IκB kinase inhibitor PS1145 (5 µM; Sigma-Aldrich, Stockholm, Sweden) at the same time as differentiation was initiated and/or after viral infection.

### Quantification of gene expression by quantitative real-time PCR

RNA isolation of cell lysates was performed using a RNA extraction kit (Nucleospin® RNA II, Macherey-Nagel, Düren, Germany). A total of 1 µg RNA was reverse transcribed to cDNA (Precision Nanoscript 2 Reverse Transcription kit, PrimerDesign, Southampton, UK) and subsequently thermocycling and real-time detection of genes of interest was performed on a Mx3005P qPCR system (Stratagene, La Jolla, CA, USA) with standard cycling parameters. Samples were analysed by ΔΔCt method^40^ and related to UBC/GAPDH expression for human-derived macrophages and to HPRT for murine-derived macrophages. All groups were normalized towards M1/M-CSF macrophage phenotype control if not stated otherwise. The following primer sequences (PrimerDesign, Southampton, UK) were used:

human IFNβ: TTACTTCATTAACAGACTTACAGGT (forward) and

TACATTAGCCATCAGTCACTTAAAC (reverse)

murine IFNβ: ATGGAAAGATCAACCTCACCTAC (forward) and

GGATGGCAAAGGCAGTGTAA (reverse)

human RIG-I: TTCTCTTGATGCGTCAGTGATA (forward) and

CCGTGATTCCACTTTCCTGAA (reverse)

murine RIG-I: CGATATTTTGAAAGACTTGGGTACA (forward) and

ATGGCTCCGTTGTTGAGATTG (reverse)

human MDA5: GTCTCGTCACCAATGAAATAGC (forward) and

TTATACATCATCTTCTCTCGGAAATC (reverse)

human TLR3: GTGTGAAAGTATTGCCTGGTTTGT (forward) and

ATGATAGTGAGGTGGAGTGTTGC (reverse)

murine TLR3: AAGTTATTCGCCCTCCTCTTGA (forward)

AGATTCTGGATGCTTGTGTTTGA (reverse)

murine IRF4: (forward) and (reverse)

HRV16: CAGAGGTTAAGAACTTGATTGAA (forward) and

CTAATTTTGTTTGTGGTGATAGAG (reverse)

### Protein expression analysis by western blotting

Samples were lysed in a lysis buffer (1% TritonX-100, 10 mM Tris-HCl, 50 mM NaCl, 5 mM EDTA, 30 mM Na_4_P_2_O_7_, 50 mM NaF, 0.1 mM Na_3_VO_4_) with the addition of protease and phosphatase inhibitors (1%). Protein content was determined and equal amounts of protein were loaded and electrophoresed on a 4–20% precast polyacrylamide gel (Bio-Rad Laboratories AB, Solna, Sweden). The fractionated proteins were blotted on a Trans-Blot Turbo Transfer System (Bio-Rad Laboratories AB, Solna, Sweden) and this was followed by blocking of the membrane in 5% (w/v) milk in Tris-buffered saline Tween-20 and overnight incubation with primary antibodies at 4 °C: anti GAPDH mAb, anti TLR3 mAb, anti RIG-I Rabbit mAb and anti MDA5 Rabbit mAb (Cell Signaling Technology, Leiden, The Netherlands). Membranes were washed and incubated for 1 h with secondary antibodies: anti Rabbit IgG HRP-linked Ab (Cell Signaling Technology, Leiden, The Netherlands). Chemiluminescent detection was performed using Clarity Max^TM^ Western ECL Substrate (Bio-Rad Laboratories AB, Solna, Sweden) and immunoblots were visualized by LI-COR Odyssey Fc Imager (LI-COR Biosciences, Lincoln, NE, USA) and Image Studio (v3.1.4; LI-COR Biosciences, Lincoln, NE, USA).

### Statistical analysis

Data are presented as mean with standard deviation. Comparison of groups was performed by Kruskal-Wallis followed by Wilcoxon post-testing (unless stated otherwise) using R^41^. P-values of less than 0.05 were considered statistically significant.

## References

[1] (GINA), G. I. F. A. *Pocket Guide for Asthma Management and Prevention*, http://ginasthma.org (2018).

[2] National Asthma, E. & Prevention, P (2007). Expert Panel Report 3 (EPR-3): Guidelines for the Diagnosis and Management of Asthma-Summary Report 2007. J Allergy Clin Immunol.

[3] Wenzel SE (2012). Asthma phenotypes: the evolution from clinical to molecular approaches. Nat Med.

[4] Byrne AJ, Mathie SA, Gregory LG, Lloyd CM (2015). Pulmonary macrophages: key players in the innate defence of the airways. Thorax.

[5] Mosser DM, Edwards JP (2008). Exploring the full spectrum of macrophage activation. Nat Rev Immunol.

[6] Mantovani A (2004). The chemokine system in diverse forms of macrophage activation and polarization. Trends Immunol.

[7] Martinez FO, Gordon S (2014). The M1 and M2 paradigm of macrophage activation: time for reassessment. F1000Prime Rep.

[8] Laza-Stanca V (2006). Rhinovirus replication in human macrophages induces NF-kappaB-dependent tumor necrosis factor alpha production. J Virol.

[9] Fong CH (2008). An antiinflammatory role for IKKbeta through the inhibition of “classical” macrophage activation. J Exp Med.

[10] Melgert BN (2011). More alternative activation of macrophages in lungs of asthmatic patients. J Allergy Clin Immunol.

[11] Hong JY (2014). Macrophage activation state determines the response to rhinovirus infection in a mouse model of allergic asthma. Respir Res.

[12] Nagarkar DR (2010). Rhinovirus infection of allergen-sensitized and -challenged mice induces eotaxin release from functionally polarized macrophages. J Immunol.

[13] Ford AQ (2012). Adoptive transfer of IL-4Ralpha+ macrophages is sufficient to enhance eosinophilic inflammation in a mouse model of allergic lung inflammation. BMC Immunol.

[14] Hershenson MB (2013). Rhinovirus-Induced Exacerbations of Asthma and COPD. Scientifica (Cairo).

[15] Alexopoulou L, Holt AC, Medzhitov R, Flavell RA (2001). Recognition of double-stranded RNA and activation of NF-kappaB by Toll-like receptor 3. Nature.

[16] Takahasi K (2008). Nonself RNA-sensing mechanism of RIG-I helicase and activation of antiviral immune responses. Mol Cell.

[17] Wark PA (2005). Asthmatic bronchial epithelial cells have a deficient innate immune response to infection with rhinovirus. J Exp Med.

[18] Korpi-Steiner NL, Bates ME, Lee WM, Hall DJ, Bertics PJ (2006). Human rhinovirus induces robust IP-10 release by monocytic cells, which is independent of viral replication but linked to type I interferon receptor ligation and STAT1 activation. J Leukoc Biol.

[19] Vogel SN, Fertsch D (1984). Endogenous interferon production by endotoxin-responsive macrophages provides an autostimulatory differentiation signal. Infect Immun.

[20] Sykes A (2012). Rhinovirus 16-induced IFN-alpha and IFN-beta are deficient in bronchoalveolar lavage cells in asthmatic patients. J Allergy Clin Immunol.

[21] Verreck FA (2004). Human IL-23-producing type 1 macrophages promote but IL-10-producing type 2 macrophages subvert immunity to (myco)bacteria. Proc Natl Acad Sci USA.

[22] Lacey DC (2012). Defining GM-CSF- and macrophage-CSF-dependent macrophage responses by *in vitro* models. J Immunol.

[23] Chanput W, Mes JJ, Wichers HJ (2014). THP-1 cell line: an *in vitro* cell model for immune modulation approach. Int Immunopharmacol.

[24] Wang N, Liang H, Zen K (2014). Molecular mechanisms that influence the macrophage m1-m2 polarization balance. Front Immunol.

[25] Wang J (2010). NF-kappa B RelA subunit is crucial for early IFN-beta expression and resistance to RNA virus replication. J Immunol.

[26] Satoh T (2010). The Jmjd3-Irf4 axis regulates M2 macrophage polarization and host responses against helminth infection. Nat Immunol.

[27] Achuthan A (2016). Granulocyte macrophage colony-stimulating factor induces CCL17 production via IRF4 to mediate inflammation. J Clin Invest.

[28] Williams JW (2013). Transcription factor IRF4 drives dendritic cells to promote Th2 differentiation. Nat Commun.

[29] Woolley KL (1994). Granulocyte-macrophage colony-stimulating factor, eosinophils and eosinophil cationic protein in subjects with and without mild, stable, atopic asthma. Eur Respir J.

[30] Obase Y (2003). Bronchial hyperresponsiveness and airway inflammation in adolescents with asymptomatic childhood asthma. Allergy.

[31] Laza-Stanca V (2011). The role of IL-15 deficiency in the pathogenesis of virus-induced asthma exacerbations. PLoS Pathog.

[32] Porta C (2009). Tolerance and M2 (alternative) macrophage polarization are related processes orchestrated by p50 nuclear factor kappaB. Proc Natl Acad Sci USA.

[33] Wathelet MG (1998). Virus infection induces the assembly of coordinately activated transcription factors on the IFN-beta enhancer *in vivo*. Mol Cell.

[34] Fleetwood AJ, Dinh H, Cook AD, Hertzog PJ, Hamilton JA (2009). GM-CSF- and M-CSF-dependent macrophage phenotypes display differential dependence on type I interferon signaling. J Leukoc Biol.

[35] Biswas SK (2006). A distinct and unique transcriptional program expressed by tumor-associated macrophages (defective NF-kappaB and enhanced IRF-3/STAT1 activation). Blood.

[36] Kawai T, Akira S (2010). The role of pattern-recognition receptors in innate immunity: update on Toll-like receptors. Nat Immunol.

[37] Rajput C (2018). Rhinovirus infection induces distinct transcriptome profiles in polarized human macrophages. Physiol Genomics.

[38] Zhu J (2019). Bronchial mucosal IFN-alpha/beta and pattern recognition receptor expression in patients with experimental rhinovirus-induced asthma exacerbations. J Allergy Clin Immunol.

[39] Newcomb DC (2008). Human rhinovirus 1B exposure induces phosphatidylinositol 3-kinase-dependent airway inflammation in mice. Am J Respir Crit Care Med.

[40] Livak KJ, Schmittgen TD (2001). Analysis of relative gene expression data using real-time quantitative PCR and the 2(-Delta Delta C(T)) Method. Methods.

[41] R: A language and environment for statistical computing (R Foundation for Statistical Computing, Vienna, Austria, 2013).

